# Replication of a Printed Volatile Mold: a novel microfabrication method for advanced microfluidic systems

**DOI:** 10.1038/s41598-019-53729-7

**Published:** 2019-11-25

**Authors:** Rémy Brossard, Thomas Brouchet, Florent Malloggi

**Affiliations:** LIONS, NIMBE, CEA, CNRS, Université Paris-Saclay, CEA Saclay, 91191 Gif sur Yvette Cedex, France

**Keywords:** Biomedical engineering, Chemical engineering, Fluidics, Biotechnology

## Abstract

A novel and simple method to fabricate microchannels is reported based on an inkjet printing of a volatile solid mold. A liquid ink -1,6 hexanediol- ejected from a piezoelectric nozzle is instantaneously frozen when touching a cooled substrate. The created mold is then poured with PDMS. Once the PDMS is crosslinked, the ink is sublimated and the device is ready. With this approach it is possible to make microchannels on different nature surfaces such as glass, paper, uncross-linked PDMS layer or non planar substrates. The versatility of this method is illustrated by printing channels directly on commercial electrodes and measuring the channel capacitance. Moreover, millimetric height microfluidic systems are easily produced (aspect ratio $\ge$ 25) as well as 3D structures such as bridges. To demonstrate, we have fabricated a combinatorial microfluidic system which makes 6 mixtures from 4 initial solutions without any stacking and tedious alignment procedure.

## Introduction

Since the invention of the first devices in the late seventies^1,2^, microfluidic technology has gained a lot of popularity. Although its impressive potential is not to demonstrate anymore, the fabrication of the device itself is still challenging on many aspects and produces a rich amount of literature. In particular, the development of a manufacturing process that satisfies the need of both academic and industrial actors is still a key issue to be addressed in order to support the development of lab-on-chip technology.

The original method of fabrication is based on glass or silicon wet or dry etching, using lithographic techniques inherited from the field of microelectronics^3,4^. It is hard to beat the remarkable accuracy of these devices. However, these methods are particularly cumbersome and costly. In particular, they require a high grade clean room and the use of expensive and hazardous chemicals.

In the beginning of the century, the invention of soft lithography^5^, supported by the commercial availability of polydimethylsiloxane (PDMS) on the market contributed greatly to the growth of microfluidics in the academic field. The technique, based on molding of structures fabricated by photolithography, is relatively simple and affordable for most laboratories. Moreover, it can be efficiently used for fast prototyping. Nowadays, it is probably the most used methods in academia. However, the fabrication of the mold is far from trivial and a controlled environment is still required. Moreover, a bonding step is still necessary to enclose the molded structure. Whereas some groups developed auto-aligning instrument for the bonding step^6^ in the majority of academic laboratories this step is still done by hand. Hence, soft lithography remains a method for specialists. Despite its great contribution, it does not seem suited for the growth of microfluidic technology in industry or in other fields. A lot of hope is put upon 3D printing technology^7–9^. Indeed, most methods based on additive manufacturing are mostly automatic, relatively inexpensive, and dedicated to 3D structures. The hope is that a non-specialist could have his chip design automatically generated by a software and directly printed. However, the resolution is still mostly unsatisfying. Typically, the channel width of printed structure with medium price setup is close to several hundred micrometers^8^. Most commercially available resins have proprietary formulations and are expensive^9^. Moreover, it is difficult to integrate additional materials, such as metal for electrodes in the final chip. This remains a major limitation of the technique. Finally, this method is very slow, as material is deposited in high resolution to print a whole block of material, immobilizing the machine for many hours. Hence, 3D printing is deemed ideal for fast prototyping but is still not suited for production. Many alternative methods have been proposed. Among those, sacrificial methods deserve some attention. The idea to use a material as a removable mold is as old as civilization, with the well-known *lost wax method*^10^ used in metallurgy. In the field of microfluidics, sacrificial material methods are not a new idea either. Since 2004, many papers proposed methods where sacrificial molds are fabricated by either lithographic techniques^11,12^ or 3D printing methods^13–15^. After embedding these structures in the future chip material, the mold has to be removed, which is usually the most problematic part of the process. The mold can be dissolved^11,13,15^, but this usually takes a very long time as it relies on the very slow process of liquid diffusion. Indeed the required time $t$ scales quadratically with the channel length $L$ following the Fick law $t\propto {L}^{2}/D$ so that the method becomes completely unrealistic for large systems: for a centimetric channel long $L=1\,cm$ and a coefficient of diffusion $D=1.1{0}^{-5}\,c{m}^{2}/s$ (diffusion of ethanol in water), the time requested to diffuse through the all channel is a day ($\sim 27h$). Other strategies have been envisioned to circumvent the problem. Sacrificial molds of polymer-based inks that can be melted were used. They can then be removed by applying pressure or vacuum at the chip opening^14,16^. However, the material is usually quite viscous and the pressure required to evacuate it from narrow channels can be very high. This strongly limits the size of the channel that can be fabricated by that method. Finally, some teams have provoked the depolymerization of the mold which can then be evacuated as gas. Polycarbonate^12^ can be used and evacuated at 450 ${}^{\circ }$C. It was successfully used to mold photosensitive polyimide. In order to lower the evacuation temperature, polylactic acid fibers doped with metallic ions can be evacuated at 200 ${}^{\circ }$C^17^. Although these process are faster, the high temperatures they require can be a limitation in a lot of application. Su *et al*.^18^ have produced the mold and the chip simultaneously by inkjet printing. More precisely, many layers of material have been printed. Printable SU8 was used where a wall was desired and soluble material was printed where a channel was wanted. This is a very tempting approach as metallic inks can also be used to add sensors in the chip. However, the fabrication is also limited by the removal of the sacrificial material and the wall material is expensive. Recently, an inkjet printing method based on the engulfing of a liquid template has been proposed by Song *et al*.^19^. Although this approach allows axisymmetric channel and fabricates systems in one step (no bonding), it is not possible to design arbitrary shapes and particularly junctions are not easy to make. Making the synthesis of previous approaches, we propose an alternative method which is simple, versatile and modular. It is based on the inkjet printing of a sacrificial material and its immersion in an appropriate cross-linkable material. We chose to use a volatile material so that it can be evacuated by evaporation quickly and at low temperature. A homemade setup has been built to implement the method and we demonstrated its numerous advantages. It is cheap, fast, trivial to use, and mostly automatic. A clean room environment is not necessary and there is no need of a bonding step. A great freedom of geometry is possible with a resolution of few tens of micrometers and using less machine time. Finally, the method has a long working distance so that it is possible to manufacture microfluidic channels elements on difficult locations, such as curved, rough or sensitive substrates. We demonstrate how this last feature can be used to include electronic or fluidic parts in the final device. Moreover, it can be used in order to assemble any kind of element without the need of standardization.

## Results and Discussion

### Fabrication process

**Figure 1:**
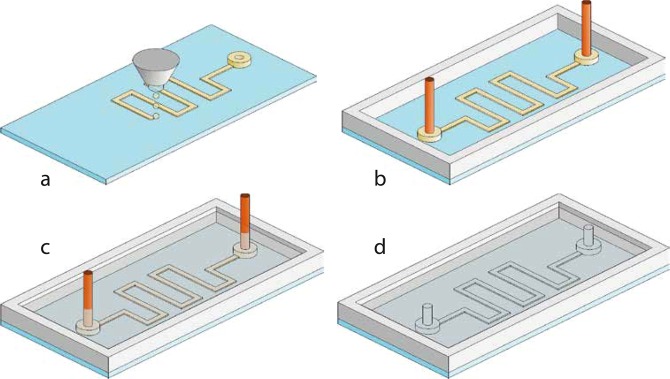
Process of fabrication of a microfluidic chip. (**a**)-Ink is deposited on the substrate in liquid phase where it freezes immediately. (**b**)-Additionnal elements can be added. Here some elements for the connections and a casing to avoid the spreading of PDMS. (**c**)-The system is immersed in PDMS which is cross-linked at a temperature below the melting point of the ink. (**d**)-The ink is evaporated, leaving open channels. The system can be used directly or removed from the substrate for further application.

The fabrication process is summed up in Fig. 1. First, the material of the mold *ie* the ink has to be deposited on a substrate in liquid phase. It should quickly solidify on the refrigerated surface to obtain a good stability of the mold. The inkjet printing method is the most suitable for this process. The method consists in generating a pattern by sending droplets of material on a substrate. Inkjet printing is a mature and very powerful technique which has been already extensively used and studied for the deposition of conductive materials^20^, polymers^21,22^ and even for tissue engineering^23^. With resolutions as low as 20 $\mu m$, it is a very versatile method as different inks can be used to produce different functions on a same substrate. At this stage, additional elements can be added on the substrate, such as a casing. A material that can cross-link is then poured on the mold, we call it the shell. The cross-linking conditions must not provoke the melting of the ink. Once the shell is solid, the channels must be opened. We evacuated the ink by evaporation. As the evacuation of gas is considerably faster than that of liquids, the time of removal of the ink is greatly reduced. To ensure a quick extraction, the evaporation temperature should be relatively low so that the removal can be completed by application of reasonable heat and vacuum. This final step is as far as we know a complete innovation.

The shell can be unstuck from the substrate and bonded on another, with plasma techniques for example. However, if the shell has a good adhesion on the substrate, the chip can be used directly. We call this method Replication of a Printed Volatile Mold (RPVM). In the present work we selected hexanediol as the ink and used PDMS as a shell material (see Methods section for more details).

**Figure 2:**
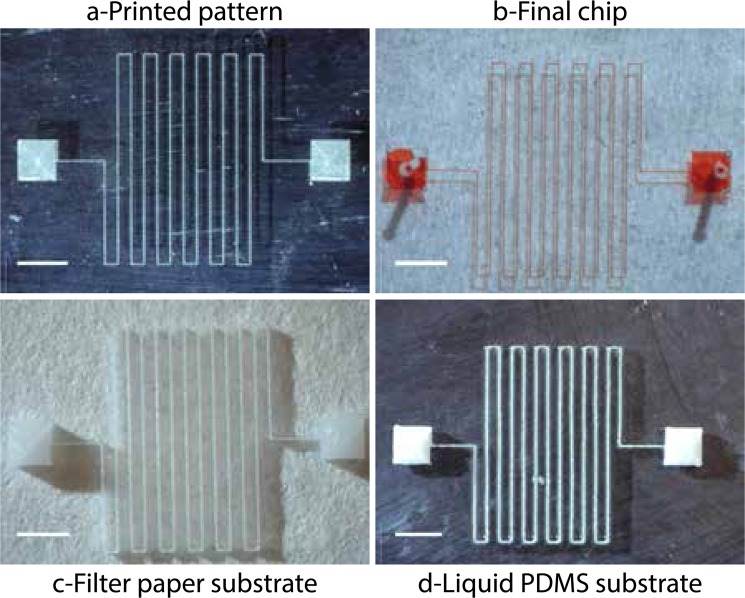
Demonstration of (**a**) hexanediol pattern printed on glass (temperature substrate 5 ${}^{\circ }$C) and (**b**) the final chip filled with red dye. The channel width is 50 $\mu$m. This chip was used directly after ink removal, ie without plasma bonding. (**c**) Non-flat cellulose acetate filter paper (temperature substrate 5 ${}^{\circ }$C). (**d**) Uncross-linked liquid PDMS (temperature substrate −24 ${}^{\circ }$C). Scale bars 2 mm.

Chips fabricated by RPVM are shown in Fig. 2. Red dye was flowed in the chip directly after the evaporation of the ink without the need of plasma bonding (PDMS directly cured on the glass). In order to test the resistance of those chips, a single channel with only one opening was printed. After molding and evaporation of the ink, the channel (190 $\mu$m wide, 180 $\mu$m height) was filled with water and put under increasing pressure. We found that the system can withhold 350mbar of pressure and breaks at 400 mbar which is sufficient for most microfluidic applications. The process can be done on hydrophilic substrate (glass and silicon wafer) as well as hydrophobic (PDMS, polypropylene, polyimide). Moreover there is no limitation on roughness or curvature for the substrate. In Fig. 2c,d a very rough filter paper, barely maintained flat, or liquid PDMS are used as substrates.

### Channel geometry

**Figure 3:**
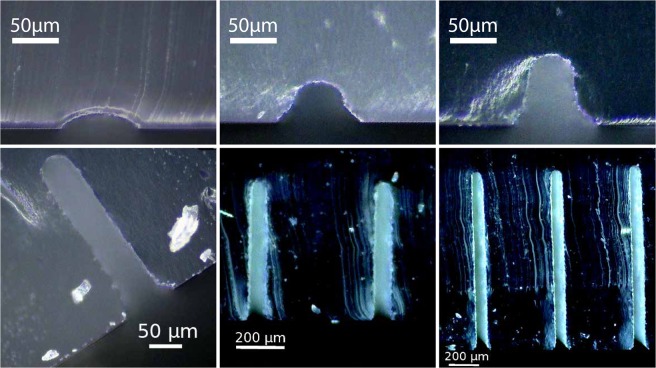
Control of channel height by printing of several layers of material. Pictures of a channel cross-section after removal of the ink. Channels were obtained by printing the same single line pattern multiple times: 1, 2, 3, 10, 20 and 40 layers were printed on top of each other.

**Figure 4:**
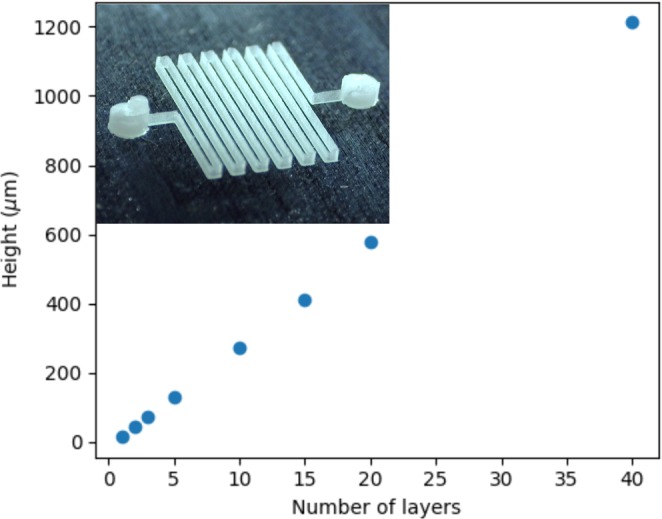
Measured height of the channel as a function of number of layers.

When only one drop line is printed, the cross-section of the channel has the shape of a lens, as can be seen in the first image of Fig. 3. The angles of the channel shape depends both of the contact angle of the ink on the substrate, of the droplet speed on impact and of the freezing time. The height of the channels can be tuned alone by printing several lines on top of each other *ie* by printing several layers of the same pattern. Vertical slices of chips printed with a different number of layers were imaged to observe the cross-section of the channels, as illustrated in Fig. 3. The first layer is relatively flat, with a width of 83 $\mu$m for a height of 16 $\mu$m. However, after the third layer, each new layer is deposited in the same manner, leading to an increase in height of 31 $\pm$3 $\mu$m for a channel width of 50 $\mu$m. It is important to note that pre-deposited layers may insulate heat transfer of the deposited ink but it seems to have no consequence for making high aspect ratio at least with a glass substrate cooled down at 5 ${}^{\circ }$C. The height of the channel as a function of the number of layers is displayed in Fig. 4 and as expected the height is linearly proportional to the deposited layers. Note that very large aspect ratios are trivially obtained, as illustrated in the inset of Fig. 4: with 40 layers a 50 $\mu$m width and $\ge$1200 $\mu$m height channel is obtained (aspect ratio $\ge$ 25) on a glass substrate at 5 ${}^{\circ }$C. This contrasts with other methods such as soft-lithography for example where the aspect ratios are more in the order of 10 due to diffraction or demolding constraints.

**Figure 5:**
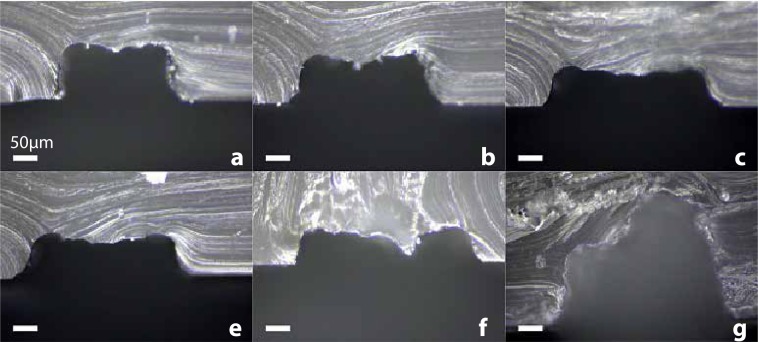
Influence of the interline length on the channel morphology. (**a**–**f**) Channels obtained by printing 3 layers of the same pattern, made of 5 parallel lines. The interline is respectively 40, 50, 60, 70 and 80 $\mu$m. (**g**) Channel obtained by printing 5 layers of 10 parallel lines with an interline of 35 $\mu$m. (scale bars 50 $\mu$m).

Similarly, the width of the channel can be tuned by printing several parallel lines next to each other. In this case, the interline *ie* the distance between each line has an influence on the height and the shape of the resulting channel, as illustrated in Fig. 5. Varying the interline between 40 and 80 $\mu$m resulted in channel heights between 75 and 110 $\mu$m. We observed a small depression at the top of most printed channel. More serious deformation can occur if the interline is too large as can be seen in Fig. 5-f. On a contrary if the interline is too small, complete distortion of the channel shape occured due to the tendency of droplets to bunch together before freezing (see Fig. 5-g). In the following of this study we fixed the line spacing at 40 $\mu$m.

### Channel roughness

**Figure 6:**
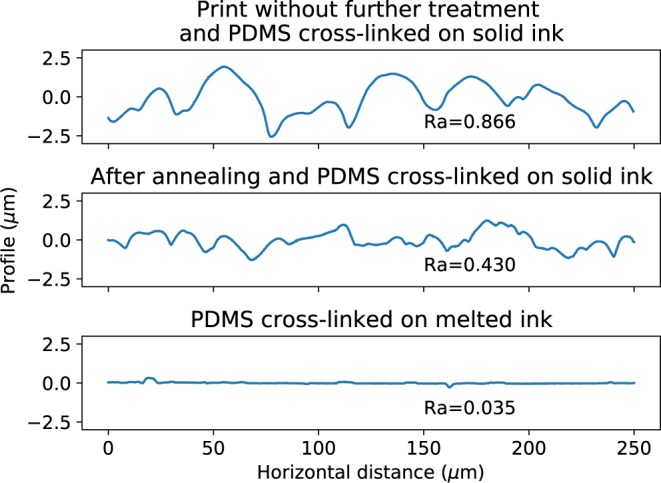
Surface roughness of fabricated channels.

The roughness of the printed structure was measured with a surface profiler as shown on Fig. 6. Different kinds of rugosity can be measured. They were characterized using the standard $${R}_{a}$$ value defined as $${R}_{a}= < | z|  > $$ where $$z$$ is the normal component of the profile, $$ < \,.\, > $$ is the average and $$| \,.\,| $$ is the absolute value. First, a freshly printed pattern has an oscillating profile which corresponds to the superposition of each droplets and is inherent to the deposition process. The associated rugosity is then $${R}_{a}=0.877\ \mu m$$.

Second, the printed structure can be smoothed by annealing in water vapor. This was obtained by bringing the freshly printed mold close to boiling water for less than a second. Because it is a very fast process, a good control could not be achieved. Nevertheless, the rugosity could be lowered without destroying the structures. The resulting profile is less dependant of the printing process, and inherent rugosity, due to the crystallinity of the material, is more visible. The associated roughness is ${R}_{a}=0.432\ \mu m$.

A final case is possible. If the printed pattern consists only in single ink layer, it is possible to melt the ink (curing temperature slightly above the melting temperature) while the PDMS is not cured yet. In the particular case of hexanediol printed on a glass substrate and covered by uncured PDMS, the triple-line appears to be strong enough to stabilize the printed pattern. As a result, it is possible to cross-link the PDMS while the ink is liquid. the shape of the patterned ink is a hemispherical lens and the resulting roughness is that of the liquid interface between the ink and the PDMS. The associated rugosity value is ${R}_{a}=0.035\ \mu m$.

### Out of the plane structures

**Figure 7:**
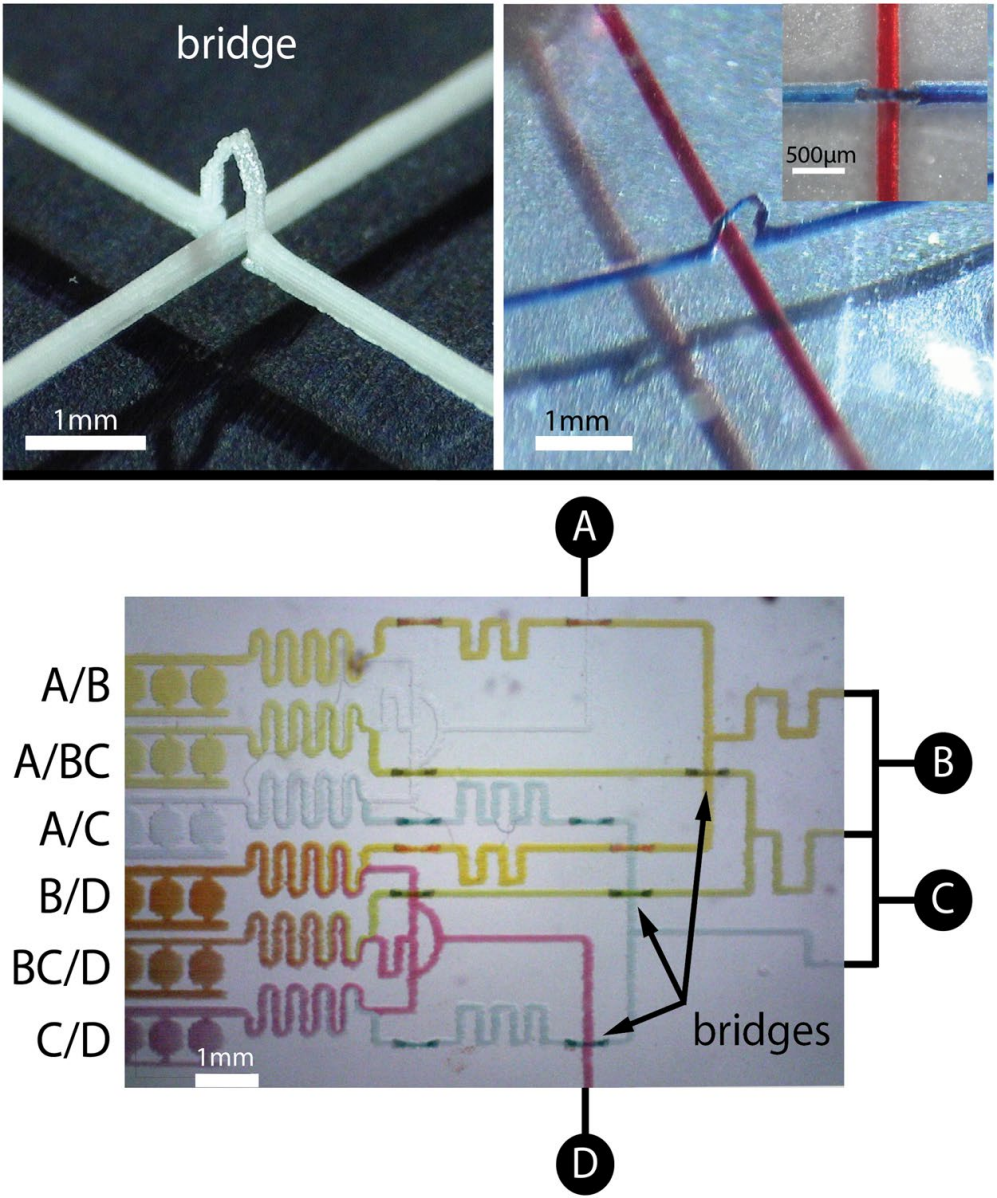
**Top**. Crossing channels mold made in a single step of printing and the resulting microfluidic chip in PDMS. The two crossing channels are filled with red and blue ink. The width of the channel is 200 $\mu$m. **Down**. Illustration of a combinatorial mixer obtained by one layer printing with the RPVM method.

In their basic form, most microfabrication methods only allow *planar* structures, designs where two channels cannot cross each other without communicating. The design has to be drawn in 2D because most 3D structures cannot be demoulded without tearing the material. An abundant literature has been generated about the many efforts to overcome this limitation for multiple purposes. Most of them rely on the fabrication of layered structures. A first method was proposed, where PDMS was sandwiched between two molds to obtain 3D structures^24^. In other processes, layers are designed separately and bonded to each other^25,26^. The use of so-called dry film^27,28^ was proposed in processes similar to multiple step photolithography. These methods are demanding both in time and equipments as they require a careful alignment of the different layers. Some alternative ideas were proposed, based on actual weaving of channels^29^ or self-repairing properties of PDMS^30^. The biggest hopes come from the field of 3D printed microfluidics, however the resolution is still limited. RPVM can overcome a lot of those difficulties. A drop will freeze in contact with another drop before touching the substrate. This phenomenon allows the fabrication of tilted structures. Two ark-like structures joined together form a bridge, and can be used for the fabrication of crossing channels (Fig. 7-Top). Combinatorial chemistry is a methodology used for screening in chemistry, material science, biotechnology and pharmaceutical fields^31^,^32^. In the microfluidic field, such combinatorial networks are made by stacking and aligning several microfluidic layers which is far from trivial^33^,^34^. Making channels that can cross each other without mixing give a substantial advantage. A proof-of-concept device providing 6 combinations (A/B, A/BC, A/C, B/D, BC/D, C/D) of 4 initial samples solution from one layer printing is demonstrated here in Fig. 7-Down.

### Integration of electronic components

**Figure 8:**
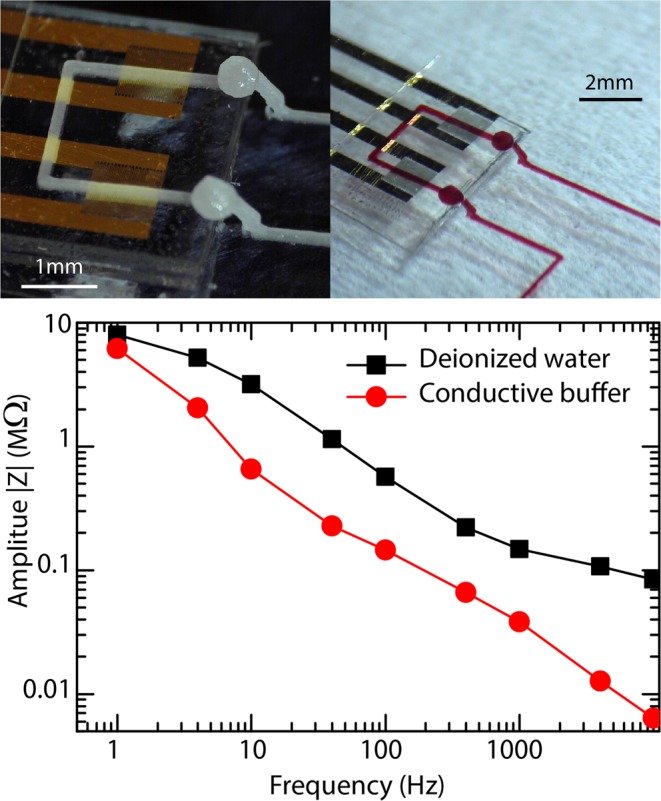
**Top**. Interdigitated gold electrodes integrated in a microfluidic channel by RPVM. **Down**. Amplitude of the complex impedance when the channel is filled with deionized water and buffer solution.

The field of microelectronics is much older than microfluidics. Miniaturized components can now be purchased at a low price and solve issues with which microfluidics groups are still struggling. Among other examples are temperature control and sensing, pressure sensing, light emission and detection, impedance probing, etc. In general, these electronic components can not be used directly in their industrial common form because the standard techniques lack versatility. Even basic assembly steps such as bringing a component in contact with a channel can be extremely difficult. For example, plasma bonding requires clean and flat substrates. One advantage of the RPVM method is that it is not necessary to be a specialist or to have access to a technical platform in order to create advanced microfluidic systems. A trivial example is the integration of electrodes described below. We purchased gold interdigitated electrodes on a glass substrate (see Methods section for more details). They are designed to be used as biosensors, for example by probing the conductivity of a solution. There is no additional space on the commercial substrate to produce a complete microfluidic chip that would integrate the electrodes. RPVM solves those problems naturally: we simply glued the glass substrate on a larger glass slide and printed channel steps on the electrodes. The device is shown in Fig. 8-Top. The complex impedance of one of the two electrodes as a function of frequency was measured when the channel is filled with either deionized water or with a solution concentrated in ions. The electrodes are made of inert gold, hence we expect to measure the capacity of an electrical double layer. As the Debye length of the concentrated solution is small, we expect to observe a larger capacity. The results are displayed in Fig. 8-Down. Although the behavior is not that of a perfect capacity, a clear difference can be seen between the two solutions and that device can be used to discriminate them.

## Conclusions

In this work, we proposed a new method based on inkjet printing of a sacrificial material that can be removed in gas phase. We call it Replication of a Printed Volatile Mold (RPVM). Hexanediol, a material which can be printed in liquid phase at 60 ${}^{\circ }$C is deposited on a cold substrate where it solidifies. PDMS is then poured on the system and cured. The hexanediol can then be removed simply and quickly by evaporation leaving open channels behind it. This last step is a great improvement compared to other methods based on sacrificial molds as the removal of the material is usually extremely long. The height of the channels can be tuned by printing several layers on top of each other. Channels with a minimal width of 50 $\mu$m and a height between 16 $\mu$m and several mm could successfully be fabricated on a broad variety of substrates. Channels made in PDMS on a glass substrate can withhold as much as 350mbar of pressure, so that plasma bonding is not required for most applications.

We showed that RPVM is extremely versatile. In particular, simple 3D structures can be realized on any substrate. We used that feature to realize non-planar design and make a complex combinatorial devices without the need of stacking. We simply integrated commercial electronic components in a microfluidic chip. This latter point in particular is of utmost interest as it allows the parallel design of a system *i.e*. components can be obtained from different sources and assembled. RPVM can be considered as the microfluidic counterpart of the printed circuit board used in microelectronics. The strength of RPVM is that it simplifies greatly the fabrication process. In particular, it does not require from end-users of other fields to become specialists of microfluidics. Thus, we believe in its great potential for the democratization of lab-on-chip technology.

## Methods

### Substrate

In this work we mainly used microscope glass slides as a substrate for printing. However, we also report some examples performed on filter paper and on uncured liquid PDMS.

### Ink

The sacrificial ink should meet a few requirements:

- it must be printable which means be in a liquid state with a viscosity lower than 30mPa.s (requirements for an inkjet system).
- it must freeze on the substrate and be stable at the cross-linking temperature of the shell material. “Stable” also means that the ink does not deteriorate in ambient conditions. For example, very hygroscopic products will melt with the humidity of the air.
- it must also chemically resist the immersion in the shell. In particular, it must have a negligible solubility with the shell material.
- it must sublimate or evaporate in favorable conditions and in a reasonable time. Typically, standard conditions accessible in the laboratory are temperature T $<$170 ${}^{\circ }$C on a hot plate at a pressure P $\approx$ 0.1bar, or room temperature at a pressure P $<$ 0.1mbar.

### Shell

The shell material should meet a few requirements:

- it must stick on the substrate after curing.
- it must not solubilize the ink.
- it must have a cross-linking temperature lower than the melting temperature of the ink.

### Choice of the material

We choose glass as a substrate and polydimethylsiloxane (PDMS) for the shell material since these two materials are widely used in microfluidics. Interestingly, because PDMS is directly cross-linked on the substrate and is never detached, we showed that its adhesion is strong enough for most microfluidic experiments. Ideally, for a practical use with PDMS, the ink melting temperature should be above 40 ${}^{\circ }$C. Regarding the previous requirements, the linear diols, with formula $(OH)-{(C{H}_{2})}_{n}-(OH)$, are good candidates for RPVM. In this work, we selected 1,6-hexanediol, which melts at 42 ${}^{\circ }$C. Its viscosity at 60 ${}^{\circ }$C is 24 mPa.s^35^ and it is insoluble in PDMS. Moreover it is cheap and biosafe. However 1,6-hexanediol is slightly hygroscopic. The setup is then put in a glove box supplied by dry air. The humidity level was maintained below 10$\%$ for the results presented here. Preliminary results show the feasibility with 1,8 octanediol with the advantage of not having to control the air humidity but all the results reported here were done with 1,6-hexanediol. Note that the thermal capacity of the ink and the heat transfer coefficient between the ink and the substrate will play an important role in the time required to freeze the ink. When the ink is not frozen quickly enough we lower the temperature of the substrate. This is the case for uncross-linked liquid PDMS for which the temperature has been lowered to −24 ${}^{\circ }$C.

### Printing setup

A homemade inket printer has been developped. The droplets were generated with a piezoelectric printhead MJ-SF-04-060 and a pulse generator JetDriveIII, both from Microfab. The position of the printhead was controlled by the C-beam 3-axis positioning system from OpenBuilds. The substrate is cooled either by a peltier or a chiller. Different temperature control elements were added both to the substrate and to the inkjet nozzle. The system was controlled using a homemade software. More details can be found in reference^36^.

### Chip fabrication

Standard microscope glass slides, rinsed with ethanol and dried with nitrogen were used as substrate. Droplets with a diameter of 50 $\mu$m of hexanediol were printed at 60 ${}^{\circ }$C at an ejection frequency of 100 Hz. Those droplets were deposited with a drop spacing of 40 $\mu$m. The height of the printed structure is tuned by printing several layers of droplets on top of each other. The temperature of the substrate was lowered to 5 ${}^{\circ }$C. The distance between the printhead orifice and the substrate surface was between 5mm and 10mm. The desired channel design was printed and the system was then left overnight at 35 ${}^{\circ }$C and 2 hours at 60 ${}^{\circ }$C in order to ensure complete cross-linking. The ink material could then be evaporated using one of the recipes of Table 1. After the ink removal, the chip is ready to use.

**Table 1:** Recipes for ink evaporation.

Time (min)	Temperature (${}^{\circ }$CC)	Pressure (mbar)
30	170	$\approx$100
120	100	$\approx$100
5,120	60, room temperature	atmospheric pressure, $<$0.1

### Integration of electronic elements

Gold interdigitated electrodes were purchased from NanoSPR and fixed on the substrate with double sided tape. The sacrificial mold was printed on the substrate and on the electrode by creating a step structure. The chip was filled with either deionized water or a conductive solution of 12080 $\mu$mScm${}^{-1}$. The complex impedance of one of the two electrodes as a function of frequency was measured with a lock-in amplifier in serie with a 1 M$\Omega$ resistance.

### Materials and reagents

Sylgard 184 (PDMS) prepolymer base and curing agent were purchased from Dow Corning (Midland, MI, USA). Hexanediol was purchased from Merck and used without purification process. Glass slides were obtained from ThermoScientific. Capillary tubes were purchased from CIL Cluzeau (France). Water was purified with an ultrapure system (Barnstead, IA, USA). The microfluidic set-up was made of a microfluidic flow control system MFCS-EZ (Fluigent, France), an Olympus CKX41 inverted microscope equipped with a pixeLINK CCD camera (pixeLINK, Canada), a DinoLite handheld USB camera (AM311-RO, AnMo Electronics Corporation, Hsinchu, Taiwan). Surface profiler is a Alpha Step stylus profiler from KLA-tencor.
